# Altered EEG Microstates Dynamics During Cue-Induced Methamphetamine Craving in Virtual Reality Environments

**DOI:** 10.3389/fpsyt.2022.891719

**Published:** 2022-05-04

**Authors:** Qianqian Lin, Dongxu Li, Cheng Hu, Zhihua Shen, Yongguang Wang

**Affiliations:** ^1^Affiliated Mental Health Center and Hangzhou Seventh People's Hospital, Zhejiang University School of Medicine, Hangzhou, China; ^2^Anhui Psychiatric Medical Center, Anhui Medical University, Hefei, China; ^3^Shiliping Compulsory Rehabilitation Center, Zhejiang, China; ^4^Zhejiang Provincial Institute of Drug Abuse Research, Hangzhou, China

**Keywords:** methamphetamine dependence, cue-induced craving, resting state, virtual reality, EEG microstates analysis

## Abstract

**Background:**

Cue-induced craving is widely considered to be the most important risk factor for relapse during abstinence from methamphetamine (Meth). There is limited research regarding electroencephalography (EEG) microstates of Meth-dependent patients under exposure to drug-related cues. Our objective was to investigate whether EEG microstate temporal characteristics could capture neural correlates of cue-induced Meth craving in virtual reality (VR) environments.

**Methods:**

EEG recordings of 35 Meth-dependent patients and 30 healthy controls (HCs) were collected during eyes-open state and cue-induced state, respectively. Group differences and condition differences in temporal parameters of four microstate classes were compared.

**Results:**

The results demonstrated the greater presence of microstate B in both Meth-dependent patients and HCs during the cue-induced condition, compared to resting state. In addition, for Meth-dependent patients, microstate C occurred significantly less frequently, along with a tendency of increased occurrence for class D during the cue-induced condition, compared to resting state. However, the change direction of class C and class D in HCs was completely opposite to that of Meth-dependent patients. The cue-induced condition also elicited different changes in transition probability between Meth-dependent patients and HCs.

**Conclusion:**

This study explored the features of EEG microstates in Meth-dependent patients during the cue-induced condition, which can improve our understanding of Meth addiction and contribute to the development of effective assessments and intervention tools.

## Introduction

Drug craving is a central concept in the research realm of addiction. Although the definition of craving is still debated (1–3), there is no controversy that drug craving is often elicited by a wide range of cues, from internal emotional changes to external drug-related cues (4, 5). Cue-induced craving is believed to be closely related to the maintenance and relapse of addictive behaviors (6, 7). Improving our understanding of the cue-induced craving would increase our knowledge of drug craving and represent potential targets for assessment and intervention.

In recent years, neuroimaging techniques have been increasingly used to identify neural correlates of cue-induced craving (8–11). Functional magnetic resonance imaging (fMRI) studies have revealed several brain networks associated with the cue-induced craving (11, 12). Inherent to the cue-reactivity paradigm, the visual processing network showed enhanced activation levels when exposed to drug-related cues (13, 14). In addition, hyperactivity of reward network and salience network were found when drug-dependent patients were confronted with drug-related cues (12, 15, 16). Somewhat counterintuitively, hyperactivity of executive network and attention network was also reported in drug-dependent patients when exposed to the drug-related cues (11, 12, 17). Since these networks both play a primary role in inhibitory control and self-regulation, the hyper-engagement of the executive and attention network suggests the recruitment of cognitive resources when they were confronting with drug-related cues (11).

Electroencephalography (EEG) is a powerful and popular method for rapid and noninvasive detection of brain signals corresponding to various states from the scalp surface area. EEG microstates, corresponding to specific EEG topographic maps, represent the global and quasi-stable neuronal activity (18, 19). It is impressive that four canonical microstate maps (i.e., labeled classes A, B, C, and D by Lehmann and colleagues) explain consistently around 80% of the total topographic variance in spontaneous EEG (20–22). This has generated much interest about the functional significance of the four EEG microstates (23–28). As expected, it has been well documented that the parameters of microstate changed in different states of consciousness (26, 28, 29), responded to external and internal stimuli (23, 24, 27), and altered in neuropsychiatric disorders [e.g., (21, 22, 30, 31)]. Although far from being conclusive, previous studies have supported the notion that class A and class B are associated with auditory network and visual network (21, 22, 24, 32), respectively, while class C and class D reflect task-negative network and task-positive network, respectively (21, 22, 24, 32).

To date, changes in the EEG microstates have been reported in addictive disorders (33–35). However, it has never been investigated the degree to which specific microstates are influenced by exposure to drug-related cues. In our previous work, Wang et al. (36) developed a methamphetamine (Meth)-related virtual reality (VR) social environment for cue-induced craving assessment. Findings indicated that exposure of Meth-dependent patients to this VR social environment could elicit an increase in heart rate variability (HRV) compared to healthy controls (HCs), and HRV is positively correlated with craving scores. Here, as a continued work, we explored for the first time the potential association between EEG microstates and cue-induced craving. Specifically, we recorded the multichannel EEG signals during VR cue-induced condition (compared to eyes-open resting condition) in Meth-dependent patients (compared to HCs).

For the inherent to the cue-reactivity paradigm, it was predicted that VR cue-induced condition would significantly increase one or more microstate parameters (i.e., duration, occurrence, and coverage) of class B for both Meth-dependent patients and HCs, which is associated with visual network. As mentioned earlier, hyperactivity of attention network was found in drug-dependent patients, reflecting by the recruitment of cognitive resources when exposed to drug-related cues. Thus, it was predicted that the cue-induced condition would significantly increase one or more microstate parameters (i.e., duration, occurrence, and coverage) of class D for Meth-dependent patients, which is associated with the dorsal attention system (21, 32) or task-positive network (22, 24). Finally, since class C is supposed to reflect the task-negative network, we also hypothesized that cue-induced condition would significantly decrease one or more microstate parameters (i.e., duration, occurrence, and coverage) of class C for Meth-dependent patients.

## Materials and Methods

### Participants

A total of 35 male participants with Meth dependence were recruited from the Shiliping Compulsory Rehabilitation Center in Zhejiang Province, China. All Meth-dependent patients had completed more than 1 month of forced detoxification. They were interviewed by an experienced clinical psychiatrist and met the following inclusion criteria: (1) they met the criteria for Meth dependence according to the Diagnostic and Statistical Manual of Mental Disorders (DSM)-IV; (2) no evidence of current or previous head injury or central nervous system (CNS) disease; (3) no history of cardiovascular disease; and (4) no other DSM-IV axis I disorder. All patients had no history of antidepressant or/and antipsychotic medication use within 2 weeks.

Notably, 30 age-matched HC male participants were recruited from the local community through advertisement. The HC met the following inclusion criteria: (1) no history of Meth use; (2) no DSM-IV axis I disorder; (3) no evidence of current or previous head injury or CNS disease; and (4) no history of cardiovascular disease.

All participants were over 18 years of age and right-handed, with normal vision and hearing. Written and informed consent was obtained. The study was approved by the local ethics committee of the Seventh Hospital of Hangzhou.

### Methods

#### Assessment Procedure

The participants were first introduced to the equipment (i.e., EEG recording device, VR helmet, and headphones). After participants felt comfortable with all the settings, a 6-min period of resting-state continuous EEG signals (i.e., eyes-open resting-state condition EEG) was recorded. Then, participants were exposed to a VR Meth-cue model (36, 37), with a concurrent recording of EEG signals (i.e., cue-induced EEG). In this VR Meth-cue model, participants were required to watch an 8-min VR video, which simulates a real Meth-related social context, including various Meth-related cues. The details of the Meth-cues VR video and its validity for craving assessment can be found in our previous study (36).

**Figure 1:**
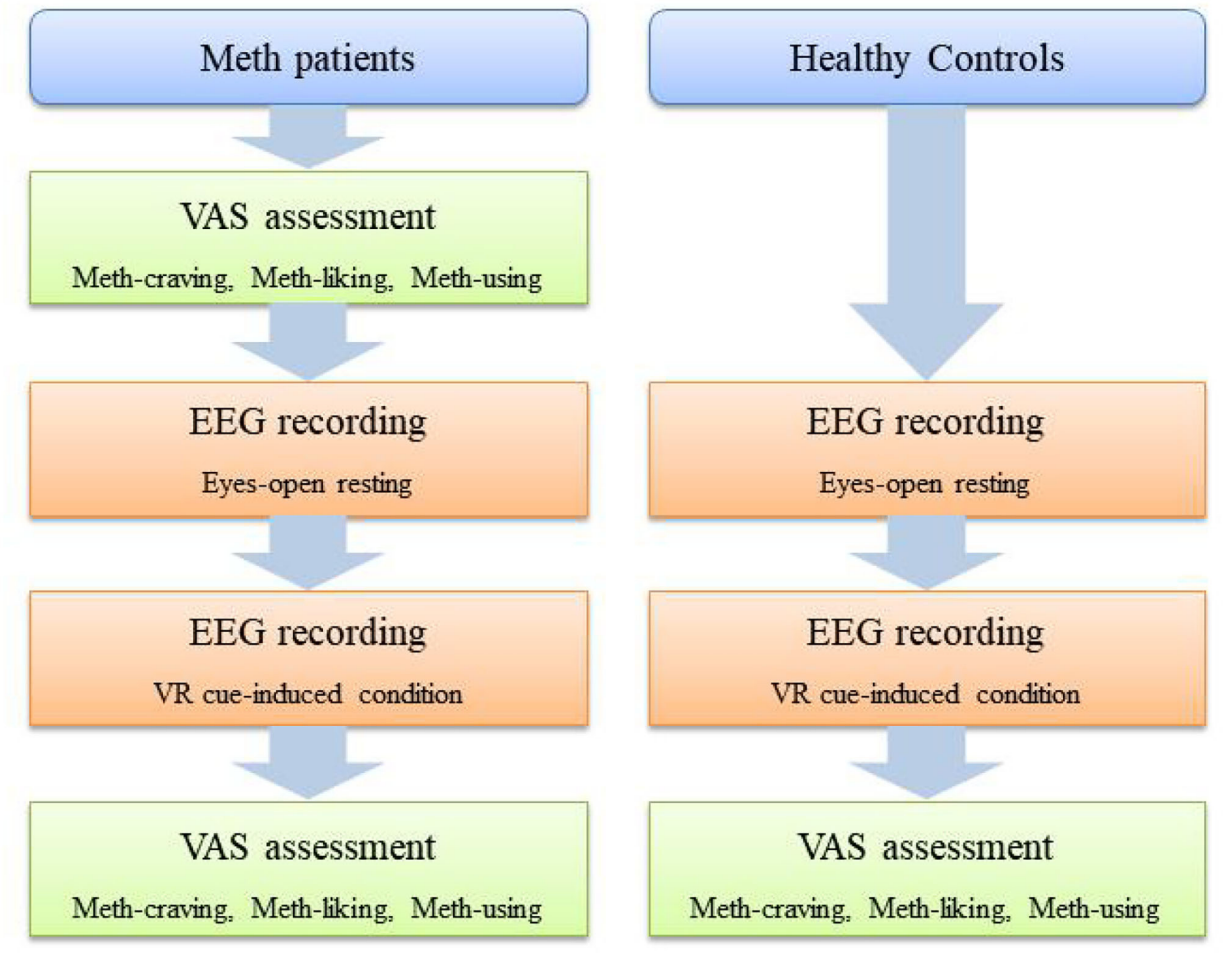
Schematic diagram of assessment procedure.

For Meth-dependent patients, before EEG recording and immediately after the VR video, they were asked to answer three questions on a visual analog scale (VAS) (i.e., VAS-craving, VAS-liking, and VAS-using). For HCs, these three VAS questions were only asked after the VR video. The schematic diagram of the assessment procedure is shown in Figure 1.

#### EEG Recording

The experiment was performed in two conditions, namely, resting condition and cue-induced condition. In the resting condition, each subject relaxed in an eyes-opened resting state as measured by EEG for 6 min. In the cue-induced condition, each subject watched an 8-min video, demonstrating Meth use with simultaneous EEG recordings. The scalp EEG data were recorded from a 32-channel EEG system according to the international 10–20 system and referenced to CPz electrode (eego^TM^ mylab, ANT Neuro, The Netherlands). The sampling rate was 2,048 Hz, and all impedances were kept below 10 KΩ.

#### VAS Assessments

Participants were asked to answer three questions on a VAS, by choosing the most suitable option for each question. The first question was regarding Meth-craving, namely, “How much do you crave Meth/ice right now?” (ranging from 0 to 10, “0” indicated “no carving at all,” and “10” indicated “extremely strong craving”). The second question was regarding Meth-liking, namely, “To what extent do you find the Meth/ice pleasant/unpleasant?” (ranging from 0 to 10, “0” indicated “very unpleasant,” “5” indicated “neither unpleasant nor pleasant,” and “10” indicated “very pleasant”). The third question was regarding the possibility of Meth-using, namely, “If you have access to Meth/ice right now, how likely would you be to use it?” (ranging from 0 to 10, “0” indicated “certainly not” and “10” indicated “certainly”).

#### EEG Data Preprocessing

Preprocessing was performed using EEGLAB version 13.0b (38) in MATLAB 2016a (The Mathwork, Inc., Natick, US). EEG data were bandpass filtered between 0.1 and 70 Hz with a Butterworth infinite impluse responses (IIR) filter, and eye movement artifacts were removed using independent component analysis (ICA). Data were subsequently segmented into artifact-free 2s epochs before microstate analysis. Bad epochs with excessive muscle activity were rejected. Then, the data were referenced to the average reference, digitally band passed at 2–20 Hz and further downsampled to 250 Hz.

#### Microstate Analysis

The EEG microstate analysis was performed separately for each group in resting or cue-induced condition. First, global field power (GFP) was computed as the spatial standard deviation of the potential field for each time point of the recording. Microstate configurations remain stable around GFP peaks, which have the highest signal-to-noise ratio and were taken as the original momentary maps (39). Original momentary maps are submitted to atomize–agglomerate hierarchical clustering (AAHC) to calculate individual microstate maps for each subject (40). The number of microstate clusters for this study was preset to four according to previous EEG microstate studies (22).

We then submitted the individual microstate maps into a second AAHC cluster analysis to identify group model maps for Meth-dependent patients with resting condition, Meth-dependent patients with cue-induced condition, HC with resting condition, and HC with cue-induced condition separately. Based on these group models, a “grand-mean” model was calculated, which was then class-labeled into microstates A-D by using minimal global map dissimilarity (GMD) (39). Next, the class-labeled “grand-mean” model maps were used as a template to assign the group model maps to the four class-labeled grand-mean maps. As a final step, the individual microstate maps were sorted into one of the microstates A-D using minimal GMD between the individual microstate maps and the class-labeled group model maps as a criterion. Four parameters were then extracted per microstate, namely, coverage (the percentage of the time covered by each microstate class), duration (the average length of time for each microstate class), occurrence (total number of each microstate class per second), and transition probability (the transition probability between any two EEG microstates). The microstate analysis was performed using EEGLAB (http://www.thomaskoenig.ch/Download/EEGLAB_Microstates/).

#### Statistical Analyses

Two-sample *t*-test and paired *t*-test were conducted to compare between groups and between conditions for age and VAS score, respectively. For the EEG microstates (i.e., coverage, occurrence, and duration), separate three-way rmANOVAs were conducted, with conditions (resting vs. cue-induced) and classes (A, B, C, or D) as a within-group factor, and groups (Meth vs. HC) as a between-group factor. For the transition probability between classes, three-way rmANOVAs were conducted, with condition and transition pairs as a within-group factor, and group as a between-group factor. *Post-hoc* multiple comparisons were conducted by family-wise error (FWE) correction.

## Results

### Demographic and VAS

Demographic data of study participants are presented in Table 1. There were no significant differences in age between Meth-dependent patients and HC. For Meth-dependent patients, the cue-induced condition had significantly higher VAS scores than the resting state, on Meth-craving (*t* = 7.604, *P* < 0.001), Meth-using (*t* = 4.866, *P* < 0.001), and Meth-liking (*t* = 3.357, *P* = 0.002). After cue-induced condition, Meth-dependent patients had significantly higher VAS scores than HCs, on Meth-craving, Meth-using, and Meth-liking (all *P*-values < 0.001).

**Table 1:** Demographic characteristics and VAS scores.

**Variables**	**Meth**	**HC**	***t*-value**	***P*-value**
	**(*n =* 35)**	**(*n =* 30)**		
Age (Years)	33.69 ± 6.46	31.57 ± 9.15	1.06	0.29
Years of Meth-use	9.83 ± 3.83			
Age of first Meth taking	24.14 ± 7.01			
**Resting condition VAS**				
Meth-craving	2.257 ± 1.559			
Meth-using	4.629 ± 3.606			
Meth-liking	3.886 ± 2.220			
**Cue-induced condition VAS**				
Meth-craving	4.457 ± 2.254	0.267 ± 0.828	10.225	<0.001
Meth-using	6.371 ± 3.388	0.400 ± 1.192	9.748	<0.001
Meth-liking	4.686 ± 1.859	1.233 ± 1.888	7.401	<0.001

### EEG Microstate Parameters

**Figure 2:**
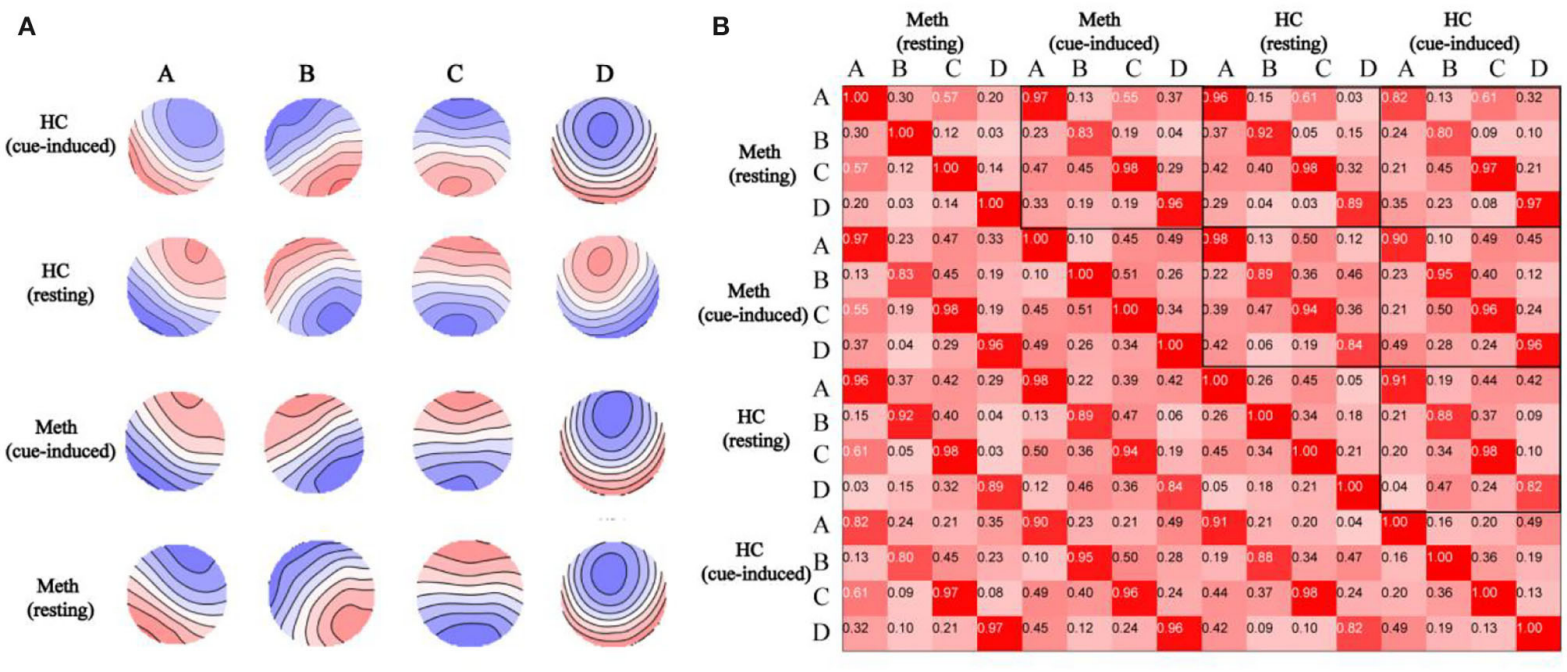
Spatial correlations are depicted between microstate topographies. **(A)** Topographies of microstate in healthy control (HCs) with cue-induced condition, HCs with resting condition, Meth-dependent patients with cue-induced condition, and Meth-dependent patients with resting condition. The topographies are labeled as microstate classes (A–D). Note that polarity (blue vs. red) is ignored in the microstate definition. **(B)** Spatial correlations between microstate topographies (A–D) identified from separate topographies of resting or cue-induced conditions in each group.

Spatial topographic correlations were used to assess between-group and between-condition similarity in microstate class topographies. Figure 2 depicts the map topographies of microstate of each group in resting or cue-induced condition and spatial topographic correlations. The four independently identified topographies were consistent with previous studies (39, 41) and were similar between conditions and groups, with mean topographic correlations exceeding 0.90.

**Figure 3:**
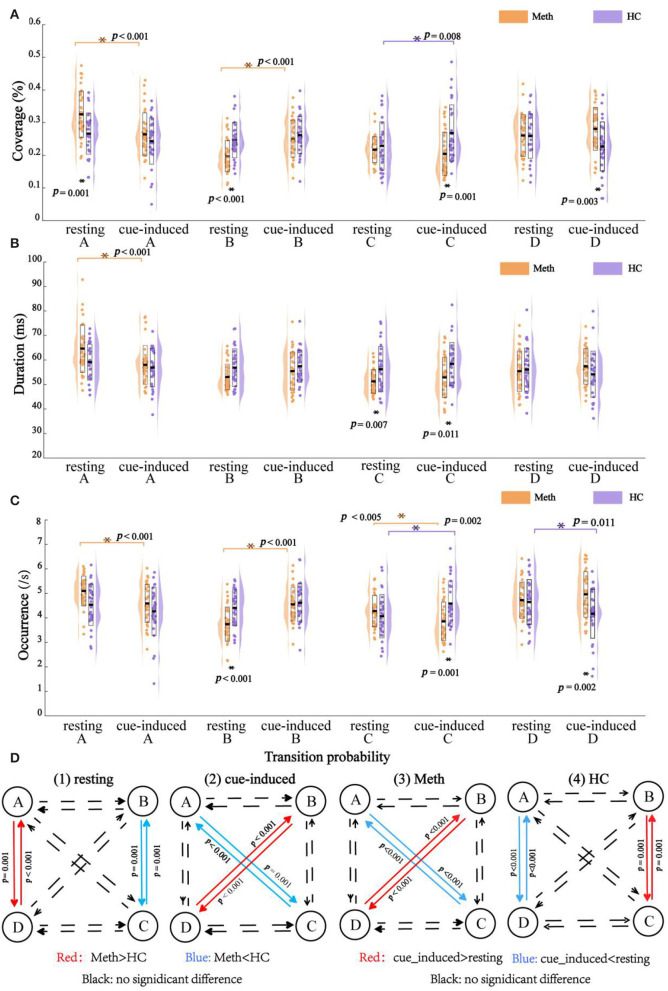
Outcomes of electroencephalography microstates with multiple comparisons. **(A)** Coverage, **(B)** duration, **(C)** occurrence, and **(D)** transition probability. Dots indicate the microstates feature and outline of violin plot represents the kernel probability density estimation. Significant *post-hoc* results between groups or between conditions are marked by asterisks.

**Figure 4:**
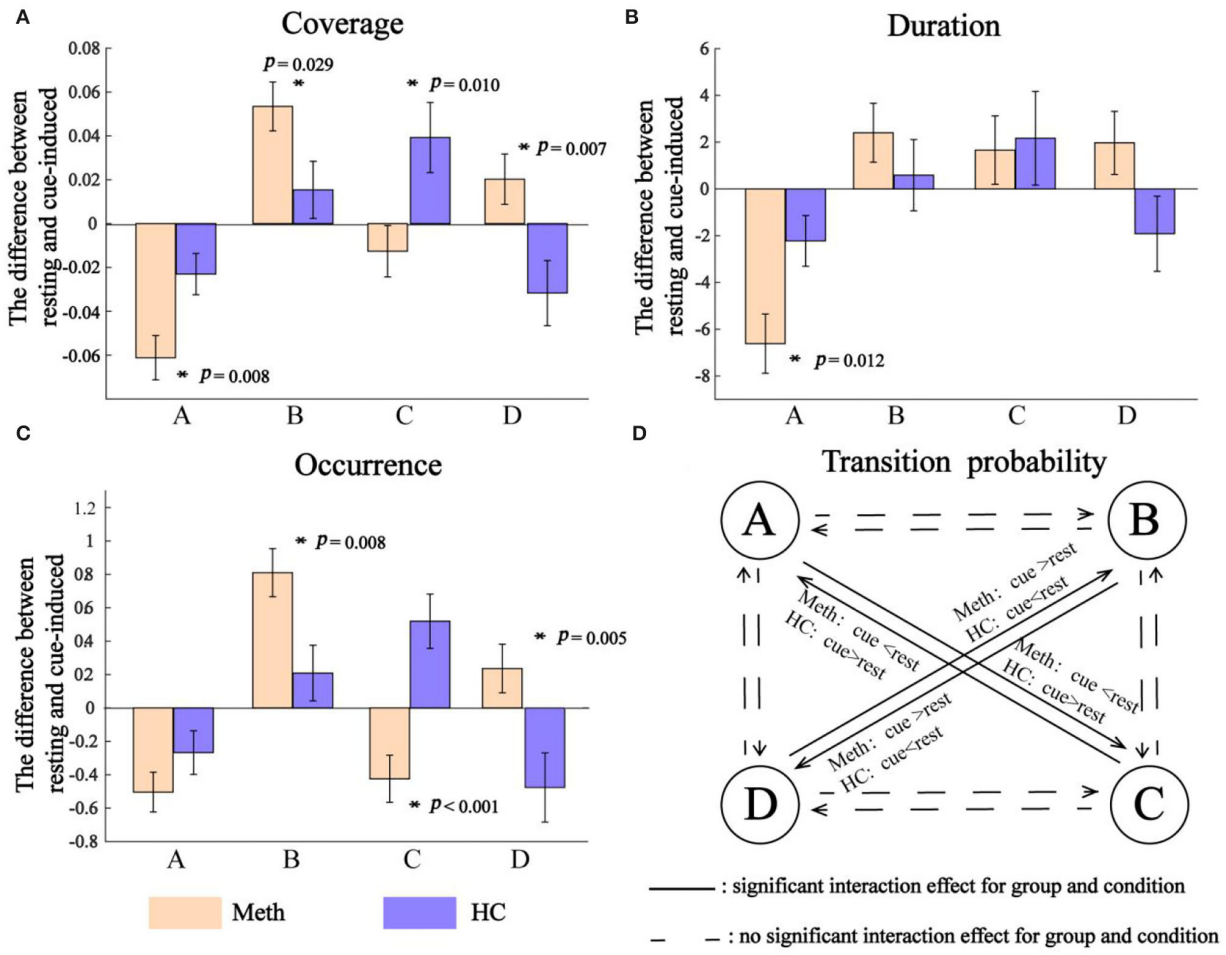
Interaction between group and condition. **(A)** Coverage, **(B)** duration, and **(C)** occurrence. Positive value indicates an increasing microstates feature from resting to cue-induced condition, while negative value represents a decreasing microstates feature. Significant interaction between group and condition is indicated by asterisks. **(D)** Transition probability. The solid line indicates a significant interaction between group and condition for transition probability of A → C, C → A, B → D, and D → B, respectively.

Outcomes of EEG microstates (i.e., coverage, duration, and occurrence) and their transition probability are shown in Figure 3. The diagram of the interaction between group and condition for each class and transition probability are shown in Figure 4.

#### Coverage

The rmANOVAs revealed a significant main effect of class (*F* = 6.495, *P* = 0.001), reflecting by class A had a larger coverage than class B (*P* = 0.010) and class C (*P* = 0.005). There was a significant interaction between condition and class (*F* = 10.490, *P* < 0.001), which was mainly explained by a decrease in the coverage of class A (*P* < 0.001), but an increase of the coverage of class B, from resting state to cue-induced condition (*P* < 0.001). No significant main effect of group and condition, and their interaction (all *P*-values = 1.000) were found. In addition, there was a significant interaction between group and class (*F* = 6.340, *P* = 0.001), which was modulated by condition (*F* = 6.870, *P* < 0.001). For the sake of simplicity, further analyses were performed for each class separately.

As shown in Figure 4, cue-induced condition elicited different pattern results across classes. For class A and class B, cue-induced condition elicited changes in the same direction for Meth and HC. There was a significant decrease of class A and a significant increase of class B in Meth (both *P-*values < 0.001), along with a decreasing tendency of class A and an increasing tendency of class B for HC. However, cue-induced condition elicited opposite changes in class C and class D for Meth and HC, respectively. Cue-induced condition elicited a decreasing tendency of class C in Meth, but a significant increase of class C in HC (*P* = 0.008). On the contrary, cue-induced condition elicited an increasing tendency of class D in Meth, but a significant decrease of class D in HC (test for interaction, *P* = 0.007). More details of multiple comparisons are shown in Figure 3.

#### Duration

The rmANOVAs revealed a significant main effect of class (*F* = 8.188, *P* < 0.001), reflecting by class A had a larger duration than class B (*P* < 0.003), class C (*P* < 0.001), and class D (*P* < 0.010). No significant main effect was found for condition (*F* = 0.190, *P* = 0.664) and group (*F* = 0.031, *P* = 0.861). There was a significant interaction between condition and class (*F* = 8.442, *P* < 0.001) (for class A, resting > cue-induced, *P* < 0.001; for class B, class C, and class D, between conditions, all *P*-values ≥ 0.121). No significant interaction was found between group and condition (*F* = 0.031, *P* = 0.861). There was a significant interaction between group and class (*F* = 6.363, *P* = 0.001), which was modulated by condition (*F* = 3.165, *P* = 0.031). For the sake of simplicity, further analyses were performed for each class separately.

As shown in Figure 4, for class A, cue-induced condition elicited a larger decrease of class A in Meth than HC (test for interaction, *P* = 0.012). No significant interaction between condition and group was found for class B (test for interaction, *P* = 0.359) and class C (test for interaction, *P* = 0.835), respectively. A marginally significant interaction between condition and group was found for class D (*F* = 3.481, *P* = 0.067). Cue-induced condition elicited an increasing tendency of class D in Meth, but a decreasing tendency of class D in HC. More details of multiple comparisons are shown in Figure 3.

#### Occurrence

The rmANOVAs revealed a significant main effect of class (*F* = 6.101, *P* = 0.001), reflecting by the occurrence of class C was significantly lower than class A (*P* = 0.024) and class D (*P* = 0.014). No significant main effect of condition (*F* = 0.083, *P* = 0.774) and group (*F* = 0.618, *P* = 0.435) was found. There was a significant interaction between condition and class (*F* = 10.969, *P* < 0.001), which was mainly explained by a decrease in the occurrence of class A (*P* < 0.001), but an increase in the occurrence of class B (*P* < 0.001), from resting state to cue-induced condition. No significant interaction was found between group and condition (*F* = 0.142, *P* = 0.707). There was a significant interaction between group and class (*F* = 6.231, *p* = 0.001), which was modulated by condition (*F* = 11.699, *P* < 0.001). For the sake of simplicity, further analyses were performed for each class separately.

As shown in Figure 4, cue-induced condition elicited different pattern results across classes. For class A and class B, cue-induced condition elicited changes in the same direction for Meth and HC. There was a significant decrease of class A and a significant increase of class B in Meth (both *P* < 0.001), along with a decreasing tendency of class A and an increasing tendency of class B for HC. However, cue-induced condition elicited opposite changes in class C and class D for Meth and HC, respectively. The cue-induced condition significantly decreased the occurrence of class C in Meth (*P* = 0.005), but significantly increased the occurrence of class C in HC (*P* = 0.002). On the contrary, cue-induced condition elicited an increasing tendency of class D in Meth, but a significant decrease of class D in HC (test for interaction, *P* = 0.011). More details of multiple comparisons are shown in Figure 3.

#### Transition Probability

The rmANOVAs revealed a significant main effect of transition pairs (*F* = 5.510, *P* = 0.002) (but pairwise comparisons, all *P*-values ≥ 0.051). No significant main effect of condition (*F* = 0.029, *P* = 0.865) and group (*F* = 1.013, *P* = 0.318) was found. There was a significant interaction between condition and pairs (*F* = 10.630, *P* < 0.001) (for A → C, C → A, A → D, and D → A, resting > cue-induced, all *P*-values ≤ 0.011; for B → C, C → B, B → D, and D → B, resting < cue-induced, all *P*-values ≤ 0.001; other comparisons between conditions, all *P*-values ≥ 0.225). No significant interaction of condition and group (*F* = 0.021, *P* = 0.885) was found. There was a significant interaction between pairs and groups (*F* = 6.094, *P* = 0.001), which was modulated by condition (*F* = 10.006, *P* < 0.001). For the sake of simplicity, further analyses were performed for each transition pair separately.

As shown in Figure 4, cue-induced condition elicited changes in transition probability between class B and class D (i.e., B → D and D → B) in the opposite direction for Meth and HC (test for interaction, all *P*-values < 0.001). Meth-dependent patients showed a significantly higher transition probability between class B and class D during cue-induced condition compared to resting state (all *P*-values ≤ 0.004), while HC showed a tendency of lower transition probability between class B and class D during cue-induced condition. In addition, cue-induced condition also elicited changes in transition probability between class A and class C (i.e., A → C and C → A) in the opposite direction for Meth and HC (test for interaction, all *P*-values < 0.001). Meth-dependent patients showed a lower transition probability between class A and class C during cue-induced condition compared to resting state (all *P*-values < 0.001), while HC showed a tendency of higher transition probability between class A and class C during cue-induced condition. More details of multiple comparisons are shown in Figure 3.

## Discussion

The goal of this study, for the first time, was to examine the effects of exposure to drug-related cues on EEG microstates under VR environment. Consistent with our hypotheses, we found that both Meth-dependent patients and HCs showed an increase in the coverage and occurrence for class B during cue-induced condition. In addition, for Meth-dependent patients, cue-induced condition elicited a significant decrease of the occurrence for class C, along with an increasing tendency of the occurrence for class D. However, for HCs, the change direction of class C and class D was completely opposite to that of Meth-dependent patients. Finally, cue-induced condition elicited a significant decrease of the A → C and C → A transition pairs in Meth-dependent patients, while HC exhibited an increased transition probability. In contrast, cue-induced condition elicited a significant increase of the B → D and D → B transition pairs in Meth-dependent patients, while HC exhibited decreased transition probability.

The data support the first hypothesis; that is, one or more parameters associated with microstate class B would increase during cue-induced condition for both groups. For Meth-dependent patients, the coverage and occurrence of class B were significantly higher during cue-induced condition compared to the resting state. For HCs, although no significant differences were found, there was an increasing tendency of the coverage and occurrence of class B, from resting state to cue-induced condition. These are congruent with findings from prior works (24, 32, 42, 43), suggesting the association between microstate class B and visual system. In addition, we found that Meth-dependent patients showed a significant decrease in the coverage, occurrence, and duration for class A from resting state to cue-induced condition. HCs also showed a decreasing tendency of the coverage, occurrence, and duration for class A from resting state to cue-induced condition. Previous studies have proposed that class A is associated with auditory system [e.g., 31, 22]. Thus, we cautiously speculate that the decrease in class A might be interpreted as the relative reduction of auditory input under VR environment. The results of transition probability between classes provide further support for our speculation. Regarding transition probability, we observed a preference for transitions to microstate B (i.e., B → C, C → B, B → D, and D → B) during cue-induced condition, compared to an eyes-open resting state. In contrast, the transitions to microstate class A (i.e., A → C, C → A, A → D, and D → A) were significantly lower during cue-induced condition, compared to an eyes-open resting state.

The findings also mostly support the hypotheses with regard to microstate class C and class D. For Meth-dependent patients, cue-induced condition elicited an increasing tendency of the occurrence for class D. In addition, for Meth-dependent patients, we also found a significantly higher transition probability between class B and class D (i.e., D → B and B → D) during cue-induced condition, compared to resting state. On the contrary, for Meth-dependent patients, cue-induced condition significantly decreased the occurrence of class C. In addition, the transition probability between class A and class C was significantly lower during cue-induced condition among patients. These findings favor the proposal that class C and class D reflect task-negative network and task-positive network, respectively (22, 24, 43). As reported by previous fMRI studies, Meth-dependent patients showed the hyperactivity of executive and attention networks under exposure to drug-related cues (11, 12, 17, 44, 45). The increase of class D and decrease of class C in Meth-dependent patients could be explained by their recruitment of cognitive resources when exposed to drug-related cues.

Most impressively, our findings revealed that HCs showed completely different result patterns regarding class C and class D. The change direction of class C and class D from resting state to cue-induced condition for HCs was opposite to Meth-dependent patients. Specifically, there were significantly higher coverage and occurrence of class C during cue-induced condition, along with significantly lower occurrence of class D. In addition, there was an increasing tendency of transition probability between class A and class C (i.e., A → C and C → A) during cue-induced condition, along with a decreasing tendency of transition probability between class B and class D (i.e., D → B and B → D). Taken together, the results with regard to class C and class D favor that “there may be a functionally relevant balance between microstates C and D, and that a preponderance of microstate C may result in a progressive detachment of mental states from environmental input” (22). Previous studies have also evidenced that relaxed, meditative, and hypnotic states were associated with an increase in class C, along with a decrease in class D (22–24, 46, 47). In addition, in our previous study (36), we also found a dissociation of the effects of drug-related cues on HRV between Meth-dependent patients and HCs. Drug-related cues induced a larger HRV for Meth-dependent patients, but a lower HRV for HCs. The relaxed, meditative, and hypnotic states, as mentioned earlier, are mostly associated with increased activity of parasympathetic nervous, which is manifested as decreasing of HRV. Taken together, the observed findings of the current work and our previous study based on HRV (36) seem toward the association between EEG microstates and HRV. This is still understudied and should be addressed in further studies.

Finally, we also found that there were significant differences between Meth-dependent patients and HCs during resting state. For example, Meth-dependent patients showed a higher coverage of class A compared to HCs, along with a lower coverage and occurrence of class B. Although the discussion of between-group differences in the resting state is beyond the scope of this study, we found that these findings are mostly inconsistent with the previous study by Chen et al. (34). In their study, they reported that Meth-dependent patients showed a lower duration of the microstate classes A and B, compared to HCs. We have speculated that this inconsistency may be related to different conditions between Chen et al.'s study (i.e., eyes-closed resting state) and the present study (i.e., eyes-open resting state). As evidenced by previous study, there were significant differences between eyes-closed state and eyes-open condition on the parameters of microstate (24).

Several limitations of this study should be noted. First, only men were enrolled in the study, and the sample size was small, which limited the generalization of results. Considering that male and female Meth-dependent patients were accommodated separately in China, multicenter and large-scale sample studies will be needed in the future. Second, to maintain context within the broader body of microstate literature, we limited our analyses to 4 model microstate classes for each experimental condition. Despite these drawbacks, our preliminary findings support that immersing patients in a Meth-related virtual social-context environment can successfully affect their EEG microstates. Further studies can build a classifier based on EEG microstate features of the significant differences between HC and Meth-dependent patients to distinguish HC and Meth-dependent patients as a potential, supplementary quantitative diagnostic tool for Meth dependence. In addition, it will be helpful to combine EEG, eye movement trajectory, and ECG, which might further deepen our understanding of the neurophysiological mechanism underlying the Meth craving.

## Data Availability Statement

The raw data supporting the conclusions of this article will be made available by the authors, without undue reservation.

## Ethics Statement

The studies involving human participants were reviewed and approved by Seventh Hospital of Hangzhou. The patients/participants provided their written informed consent to participate in this study.

## Author Contributions

QL contributed to data collection, data analysis, writing of original draft, manuscript redaction, and revisions. DL and CH contributed to data collection. ZS contributed to the study design. YW contributed to study design, manuscript redaction, and revisions. All authors contributed to the article and approved the submitted version.

## Funding

This work was supported by the Science and Technology Bureau of Hangzhou (Grant Nos. 20190101A11 and 20201203B190), the Zhejiang Provincial Natural Science Foundation of China (Grant Nos. LGF18H090023 and LGF22H090036), and the Project for Hangzhou Medical Disciplines of Excellence and Key Project for Hangzhou Medical Disciplines.

## Conflict of Interest

The authors declare that the research was conducted in the absence of any commercial or financial relationships that could be construed as a potential conflict of interest.

## Publisher's Note

All claims expressed in this article are solely those of the authors and do not necessarily represent those of their affiliated organizations, or those of the publisher, the editors and the reviewers. Any product that may be evaluated in this article, or claim that may be made by its manufacturer, is not guaranteed or endorsed by the publisher.
